# Handwork vs machine: a comparison of rheumatoid arthritis patient populations as identified from EHR free-text by diagnosis extraction through machine-learning or traditional criteria-based chart review

**DOI:** 10.1186/s13075-021-02553-4

**Published:** 2021-06-22

**Authors:** T. D. Maarseveen, M. P. Maurits, E. Niemantsverdriet, A. H. M. van der Helm-van Mil, T. W. J. Huizinga, R. Knevel

**Affiliations:** Department of Rheumatology, Leiden University Medical Center, Leiden, The Netherlands

**Keywords:** Rheumatoid arthritis, Machine learning algorithms, EHR, Electronic health records, Artificial intelligence, Classification criteria, Big data, Observational research, Chart review

## Abstract

**Background:**

Electronic health records (EHRs) offer a wealth of observational data. Machine-learning (ML) methods are efficient at data extraction, capable of processing the information-rich free-text physician notes in EHRs. The clinical diagnosis contained therein represents physician expert opinion and is more consistently recorded than classification criteria components.

**Objectives:**

To investigate the overlap and differences between rheumatoid arthritis patients as identified either from EHR free-text through the extraction of the rheumatologist diagnosis using machine-learning (ML) or through manual chart-review applying the 1987 and 2010 RA classification criteria.

**Methods:**

Since EHR initiation, 17,662 patients have visited the Leiden rheumatology outpatient clinic. For ML, we used a support vector machine (SVM) model to identify those who were diagnosed with RA by their rheumatologist. We trained and validated the model on a random selection of 2000 patients, balancing PPV and sensitivity to define a cutoff, and assessed performance on a separate 1000 patients. We then deployed the model on our entire patient selection (including the 3000). Of those, 1127 patients had both a 1987 and 2010 EULAR/ACR criteria status at 1 year after inclusion into the local prospective arthritis cohort. In these 1127 patients, we compared the patient characteristics of RA cases identified with ML and those fulfilling the classification criteria.

**Results:**

The ML model performed very well in the independent test set (sensitivity=0.85, specificity=0.99, PPV=0.86, NPV=0.99). In our selection of patients with both EHR and classification information, 373 were recognized as RA by ML and 357 and 426 fulfilled the 1987 or 2010 criteria, respectively. Eighty percent of the ML-identified cases fulfilled at least one of the criteria sets.

Both demographic and clinical parameters did not differ between the ML extracted cases and those identified with EULAR/ACR classification criteria.

**Conclusions:**

With ML methods, we enable fast patient extraction from the huge EHR resource. Our ML algorithm accurately identifies patients diagnosed with RA by their rheumatologist. This resulting group of RA patients had a strong overlap with patients identified using the 1987 or 2010 classification criteria and the baseline (disease) characteristics were comparable. ML-assisted case labeling enables high-throughput creation of inclusive patient selections for research purposes.

**Supplementary Information:**

The online version contains supplementary material available at 10.1186/s13075-021-02553-4.

## Introduction

Electronic health records (EHRs) contain a vast amount of observational data. Manual review of these data is time-consuming and laborious, hampering the usability of the data. Advancements in the Natural Language Processing and machine learning (ML) methods have created great potential for processing format-free text data such as present in EHRs [1, 2]. These EHR entries contain the prosaic conclusion of the treating physician, ranging from elaborate discussion of lab results to listed differential diagnoses. This unstructured nature of the records makes them hard to query using simple text matching. Machine learning methods can deduce patterns from a set of training examples, without requiring any domain-specific knowledge. The algorithm does not count specific criteria, but rather identifies discriminatory features by learning from an annotated outcome. We have previously developed a pipeline for the identification of rheumatoid arthritis (RA) in format-free text from clinical notes of the rheumatology clinic [3]. The machine learning pipeline generates algorithms (i.e., classifiers) by which we can extract the RA patients from 17,662 rheumatology EHRs in merely 5 s with high confidence. These algorithms recruit words and chunks in the text as features to identify the RA-cases with respect to the final diagnosis of the rheumatologist which is treated as the true label [4]. Hereby, we ensured capturing even patients diagnosed with RA, for whom the rheumatologist did not register the components of the 1987 or 2010 EULAR/ACR RA criteria.

Now the question arises whether the patient selection of our high-throughput machine learning approach, which is steered by rheumatologist’s diagnosis, differs from the traditional manual chart review, which uses classification criteria as golden standards.

In the current descriptive study, we compared the patients selected by our machine learning pipeline to patients selected through (traditional) manual chart review applying the ACR/EULAR 1987 and 2010 criteria.

## Method

### Patients

We retrieved the records of all patients who visited the Leiden University Medical Center (LUMC) rheumatology clinic between the initiation of the EHR system in 2011 and 2019 (n=17,662). From these dossiers, we used the “Conclusion” section of the physician’s notes, which describes the symptoms and differential diagnosis. Using a support vector machine (SVM) model we built a classifier, capable of extracting the rheumatologist’s RA diagnosis from these unstructured data. We randomly selected 3000 patients and asked a rheumatologist to review the medical records. Patients were annotated purely on the diagnosis of their own rheumatologist after 1 year of follow-up. To develop our ML model, we created two distinct datasets: 2000 patients were selected to train and validate the model and the remaining 1000 patients were set apart as an independent test set to evaluate model performance. The SVM was selected as the best fit to our data by the pipeline presented in Maarseveen et al. 2020, outperforming various other models, such as a neural network and a rule-based query [4].

The SVM identifies these diagnoses by finding the optimal boundary (hyperplane) separating the different classes (RA and non-RA) using both individual words and chunks of sentences as features. The most discriminatory features that contribute to the SVM’s decision can be found in previously published work [4]. It employs the kernel trick, where it maps the samples into a higher dimensional space in order to find the hyperplane [5]. The output of the classifier is a score from 0 to 1, where scores represent the likelihood of a patient having RA. The cutoff for binarization (RA yes/no) can be tweaked depending on the need for a particularly precise or sensitive patient selection.

A subset of the patients from the EHR was also included in an observational cohort. Patients presenting at the LUMC rheumatology clinic with arthritis are asked to participate in this cohort, which comprehensively registers a wide variety of medical data [6]. In 1993, this population-based prospective cohort started collecting patient information every 3 months in the first year of patient follow-up and after that yearly. Inclusion took place when arthritis was confirmed at physical examination and symptom duration was <2 years. The final diagnosis was obtained at a 1-year follow-up by manual chart review of one rheumatologist who counted the 1987 and 2010 EULAR/ACR criteria in patients that were diagnosed with RA [7, 8].

Ethical consent was obtained from the ethics committee of the LUMC before the initiation of the study.

So for the current study, we created a dataset that contained patients who visited the outpatient clinic for the first time between 2011 and 2019 and who were manually checked for fulfilling the 2010 and 1987 classification criteria as part of their enrollment in our early arthritis cohort. To this set of patients, we applied three methods to identify RA: the SVM model extracting the rheumatologist’s diagnosis, the 1987 classification criteria, and the 2010 classification criteria.

### Statistical analyses

We describe the model which resulted from training an SVM algorithm on the medical records of 2000 randomly selected patients in previously published work [4]. The final threshold for ML case identification was set by optimizing the trade-off between positive predictive value (PPV) and sensitivity in the training set. To test whether the model was robust, we evaluated the performance of the SVM-derived classifier (sensitivity, specificity, PPV, and negative predictive value (NPV)) using the diagnosis of the treating rheumatologists as a gold standard. In addition, we visualized the performance by rendering an ROC and PR curve with Scikit-learn package v0.21.2 in Python v3.5 [9].

Next, we examined the extent of overlap between the patients identified with the machine learning classifier and those identified using the criteria approach. The relationship between the cohorts was visualized in an upset plot with the R UpSetR package v1.4.0 in R v4.0.2 [10]. Finally, we compared the baseline demographics and disease characteristics of the different RA-case selections using Pearson chi-squared and Mann-Whitney U tests (α = 0.05).

**Fig. 1:**
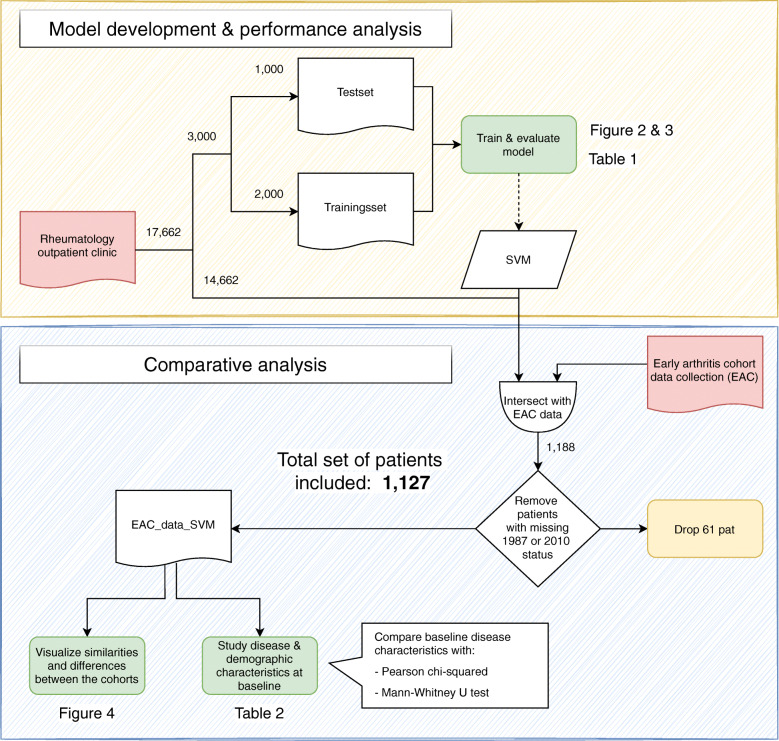
Study workflow depicting the model development and evaluation procedure (orange section) and the criteria comparison analysis (blue section), whereby the important analysis-steps are highlighted in green. We ran the SVM identification on all 17,662 patients of the Rheumatology outpatient clinic. Next, we selected only those patients that were also included in the EAC (n=1188) and who were annotated for the 1987 and 2010 criteria, resulting in a final selection of 1127 patients for the final analysis. The patient collections are indicated by a wave line box, whereby the initial two data sources are colored red (rheumatology outpatient clinic = patients from the Leiden outpatient clinic with the first consult after 2011; early arthritis cohort = research cohort patients with the first consult after 2011).

Figure 1 describes the flow of case selection and tests of our study.

## Results

### Performance of ML in identifying the RA cases as diagnosed by the treating rheumatologist

**Fig. 2:**
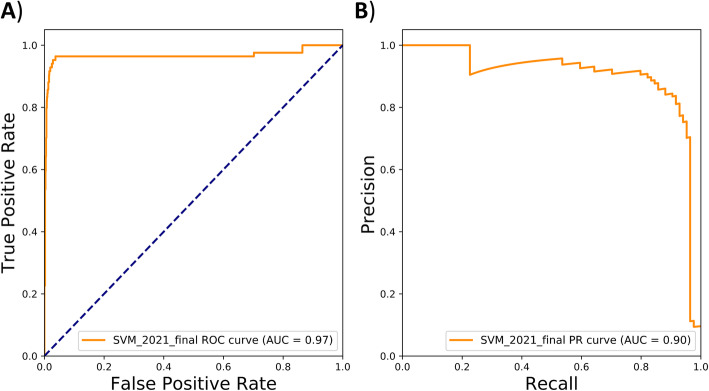
**A** Receiver operating characteristics plotting the sensitivity against the specificity and **B** precision-recall curve plotting the positive predictive value (precision) against the sensitivity (recall) for the support vector machine classifier in the independent test set. The precise features (top 20) that constitute the support vector machine model can be found in the original study by Maarseveen et al (2020 )[4]

The flexible nature of the SVM binarization cutoff (RA yes/no) enables us to choose a very precise, very sensitive, or a balanced approach to the performance of the algorithm (Table 1 and Figure S1). To make sure we find the largest number of definite cases, we took a balanced approach between PPV and sensitivity of the SVM, which resulted in a probability cutoff of 0.83 based on our training data. We then applied this cutoff to the independent set of 1000 annotated patients. In this set, the SVM-based ML classifier had an AUC-ROC and AUC-PRC of 0.97 and 0.90 respectively (Fig. 2). The classifier performed very well at identifying patients that were diagnosed with RA by their rheumatologists: sensitivity 0.85, specificity 0.99, PPV, 0.86, and NPV 0.99 (Table 1).

**Table 1:** Performance characteristics for different cutoffs of the SVM ML RA identification score in the independent test set

	0.53	0.83	0.99
Sens	0.93	0.85	0.71
Spec	0.97	0.99	1.00
PPV	0.75	0.86	0.94
NPV	0.99	0.99	1.00

*ML* machine learning, *SVM* support vector machine, *RA* rheumatoid arthritis, *PPV* positive predictive value, *NPV* negative predictive value

### The extent of overlap between machine learning and criteria-based selections

A total of 17,662 novel patients visited the Leiden outpatient clinic since the EHR initiation in 2011. In this set, the ML identified 1318 patients with a diagnosis of RA by their rheumatologist after 1 year of follow-up. In the same period, the prospective cohort included 1188 patients with early arthritis. Patients in whom the 2010 and 1987 criteria were not assessed at all were excluded, leaving 1127 patients for this paper’s analyses (Fig. 1).

**Fig. 3:**
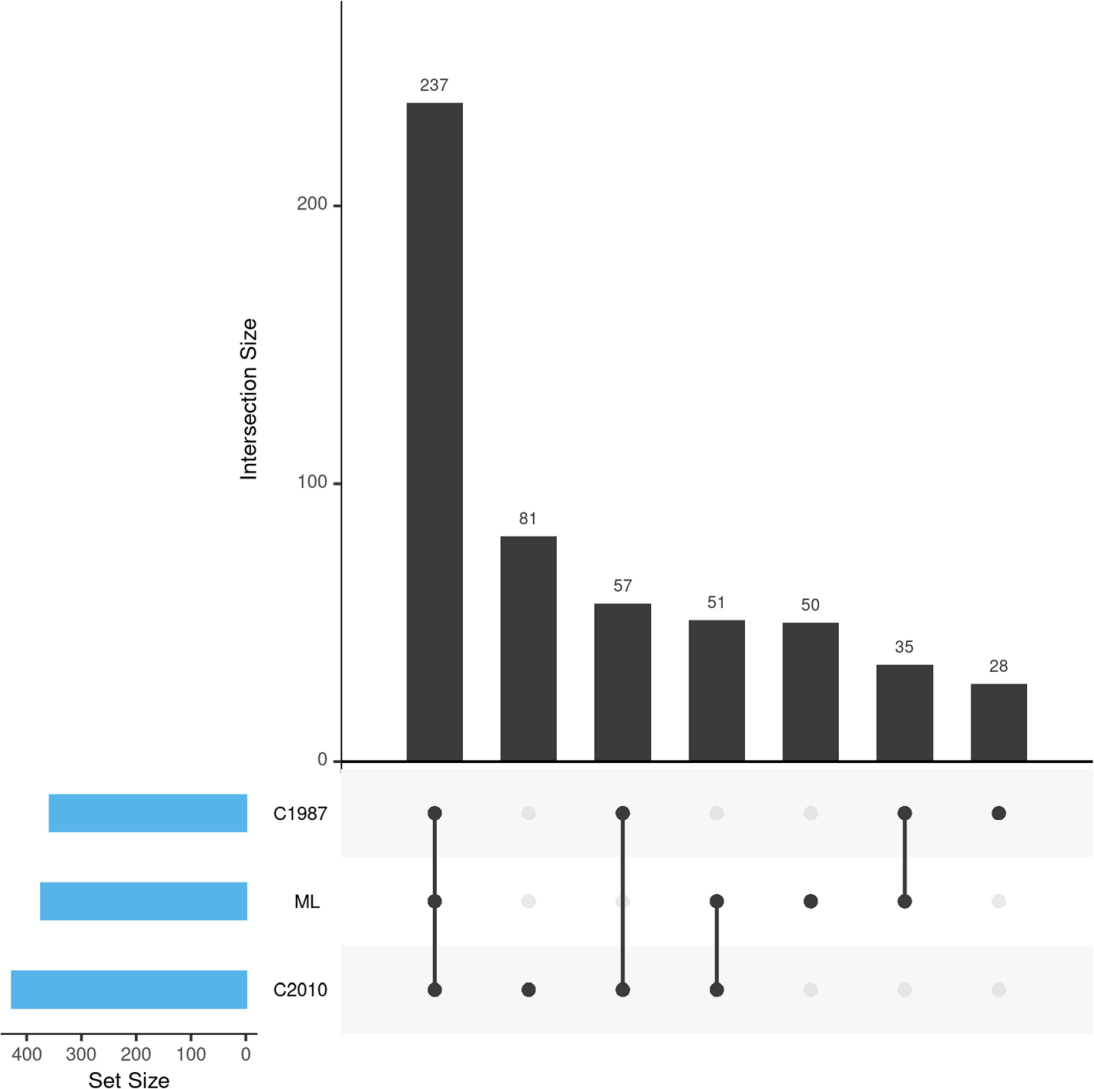
Upset plot visualizing the intersections of the ML-defined cohort and the 2 criteria-based selections, with a bar chart depicting the total cohort size in the bottom-left where C1987 = 1987 criteria-based cases, ML = machine learning-based cases, and C2010 = 2010 criteria-based cases. N = 539 unique cases out of 1127 records

To visualize the overlap of the ML-defined RA cases to the 2010 and 1987 RA criteria selections, we rendered an upset plot (Fig. 3). In our set of 1127 patients with both EHR data and criteria-based annotation 539 unique RA cases were identified. Of these, 373 (69.2%) were identified by our ML as having RA. In the same set, 426 (79.0%) fulfilled the 2010 criteria and 357 (66.2%) the 1987 criteria. The overlap between the different selection methods was substantial: 237 (44.0%) were identified with all three methods, and an additional 86 (16.0%) were identified by both ML and one of the classification criteria (51 (9.5%) and 35 (6.5%) for 2010 and 1987, respectively). The ML identified 50 (9.3%) patients for whom all classification criteria were assessed, but who were negative on both sets, whereas 81 (15.0%) and 28 (5.2%) patients met a single classification criteria set (2010 or 1987, respectively) and were not identified by the ML. A final group of 57 (10.6%) patients met both classification criteria but not the ML cutoff. The ML-defined set had slightly more overlapping patients with the 2010 criteria than the 1987 criteria (288 (53.4%) and 272 (50.5%), respectively).

### Demographic and baseline differences in machine learning and criteria based selections

In Table 2, we compared the baseline characteristics of the RA cases identified by ML to the patients fulfilling the two sets of criteria. The group of patients that was diagnosed with RA by their rheumatologists had the same median age, DAS44 at baseline, prevalence of women, anti-CCP-positivity, and RF-positivity as patients selected based on fulfilling the 2010 or 1987 classification criteria. We found no statistically significant differences between the three groups.

**Table 2:** Comparison of baseline characteristics between the machine learning defined case selection (cutoff=0.83) and the two criteria based selections

	Patients from the cohort with EHR data and classification data		
	Predicted case based on machine learning (cutoff=0.83)	1987 criteria Based cases	2010 criteria Based cases
N^☨^	373	357	426
Proportion women	0.65	0.63	0.66
Proportion anti-CCP2-positive	0.52	0.49	0.49
Proportion RF-positive	0.56	0.57	0.58
Median DAS44 at baseline	2.8	2.9	2.9
Median BMI	26.0	25.6	25.6
Median ESR	25	29	27
Median CRP	9.5	10.2	9.0
Median age at inclusion	57.2	58.6	57.2
Median symptom duration at diagnosis (days)	92.0	90.0	91.0
Median number of swollen joints	5	6	6

P values were calculated with the Pearson chi-squared for proportions, Mann-Whitney U for medians: *p<0.05; **p<0.01, ***p<0.001; ^☨^*Not statistically tested*

### Description of patients exclusively found by either the ML or criteria

To further elucidate the cases exclusively identified by the ML and those exclusively identified by the criteria, we investigated the baseline characteristics for these subgroups as well (Table 3). The ML identified 50 patients who were not found by the criteria. This group had an abundance of seronegative scoring patients, with a CCP-positivity of 7% and a RF-positivity of 17%, respectively. The criteria-based approach identified 166 patients that were not found by the ML. The majority of cases that were only found by the criteria were also anti-CCP2- and RF negative: 16% and 34%, respectively. There were no clear differences with regard to other patient characteristics.

**Table 3:** Baseline characteristics of the cases exclusively identified by the ML and those exclusively identified by either the 1987 or 2010 criteria

	Only-ML not criteria	Only-criteria not ML
N☨	50	166
Proportion women	0.60	0.63
Proportion anti-CCP2-positive	0.07	0.16
Proportion RF-positive	0.17	0.34
Median DAS44 at baseline	2.52	2.74
Median BMI	26.6	25.6
Median ESR	19.0	25.0
Median CRP	9.35	8
Median age at inclusion	55.7	58.9
Median symptom duration at diagnosis (days)	66	78.0
Median number of swollen joints	4.0	4.0

### Upset and baseline table for different cutoffs

In addition to the balanced cutoff of the ML probability, we studied the effect of a more stringent and a more lenient cutoff. The ML with the stringent cutoff (0.99) was, as expected, much more precise, but less sensitive (Table 1). With this cutoff the ML identified 282 patients (Table S1), 195 of those overlapped with both 1987 and 2010 criteria selections (Figure S2). This group of patients had a similar age, prevalence of women, and RF-positivity as the criteria-based selections. The anti-CCP-positivity prevalence (58%) was substantially higher compared to both the 1987 (*P*=0.022) and 2010 criteria (*P*=0.016).

With the lenient cutoff of 0.53, the ML was very sensitive but less precise (Table 1). Here, we identified 428 patients (Table S2) of which 248 patients fulfilled both criteria (Figure S3). The group of ML-identified cases maintained a similar prevalence of women, RF-positivity, and anti-CCP-positivity as those who fulfilled one or both of the two classification criteria. We did, however, find substantial differences in the disease characteristics. The median number of swollen joints (5) was significantly lower with respect to the 1987 criteria-based selection (*P*=0.022). Notably, this p value would not survive a correction for multiple testing.

## Discussion

Our study describes the production and validation of a robust machine learning model that extracts high-quality patient collections from free written EHR data. By extracting the diagnosis of the treating rheumatologist, we are able to confidently classify patients as RA cases even when information on classification components is missing. Our method is both fast and efficient (3326 complete medical records per second) and creates a highly similar case selection to criteria-based chart review.

Traditionally, researchers use the classification criteria for the creation of their datasets in order to select a homogenous patient cohort. While these criteria are rigorously validated and generally accepted by the community, they are by no means a replacement for the clinical judgment of a rheumatologist [11–13]. Defining what constitutes a true RA case has always been the prerogative of the expert community of rheumatologists. The aim of both the 1987 and the 2010 criteria is the inclusion of said cases, with a preference for more stringent selection over larger, more heterogeneous cohorts. As a result, the general population of RA patients extends beyond the scope of the classification criteria. Many research ventures (e.g., drug trials) align with the inclusion of criteria positive patients (high specificity), as their effects should not be diluted by noisy patient populations. However, when we are interested in investigating or redefining the entire disease entity of RA, a broad inclusion is much preferred (high sensitivity). In these latter instances, the rheumatologist diagnosis is the ideal balance between inclusiveness and precision. The EHR format-free text fields filled out by the treating physician are the most extensive collection of these diagnoses, but these are very time and resource consuming to manually peruse. By using high throughput machine learning approaches to crunch outpatient clinic EHRs, it is feasible to classify thousands of patients based on their rheumatologist’s diagnosis in mere seconds. We show that machine learning can be employed to empower clinical studies by unlocking the wealth of information in the EHR. Patients simply have to provide access to their EHR records. No additional visits are required, thus reducing the burden on patients. Thereby, we can also include patients who would be unable or unwilling to enter a research cohort or who missed the inclusion because it was not offered to them, which results in a reduced risk of selection bias. The quick cohort generation of homogenous subpopulations facilitates both clinical and translational research. For example, we could leverage the EHR records to elucidate subgroups of patients to advance the precision medicine field.

Between 2011 and 2019, 17,662 patients visited the Leiden outpatient clinic. In total, we identified 1318 patients diagnosed with RA in the outpatient clinic, 373 of whom were present in the observational arthritis cohort we used in this study. This higher patient number could increase study power. However, while the ML method increases sensitivity, it slightly reduces precision when one considers fulfilling classification criteria the golden standard. Though manual chart review searching for individual criteria will also have its imprecisions, it might hit a better balance between precision and sensitivity than our probabilistic approach. Furthermore, there is a great benefit to data that is registered into the consistently structured prospective cohorts by specialized research personnel. On the other hand, EHRs often contain a larger quantity and higher variety of data whose collection is not constrained by a specific study design. Using these data for research purposes will require stringent data curation. This curation step involves the manual annotation of a fraction of the data. Fortunately, natural language processing and machine learning make the wealth of (noisy) EHR data more accessible than ever. The pipeline solely requires a small annotated set to train and validate a qualitative model which can in turn be deployed on the entire data. Regardless, depending on one’s research question, either structured cohorts or EHRs will be more suited for data collection.

We show that our ML constructed patient selection is highly comparable to the patient groups meeting the 1987 and 2010 criteria. Nevertheless, we also identify 2 groups of single-positive patients; those who are diagnosed with RA by their rheumatologist, but who do not meet either of the criteria sets, as well as those who meet one or both of the criteria, without officially being diagnosed. When looking at the clinical characteristics of these groups, it becomes apparent that these are both composed of patients with for example a much lower prevalence of anti-CCP and RF. The single positives therefore seem quite divergent from the double positives and those caught extra by one method and seem to balance out well with those missed according to the other.

One might think that ML methods are not truly required to deal with the extensive information in the EHR data; the use of standardized billing codes for the purpose of case identification requires only simple queries. However, previous work has shown a tendency for these codes to identify false positives, with up to 33% of identified cases not representing true RA diagnoses in this same population [14, 15]. The clinical gold standard diagnosis of a treating rheumatologist is undoubtedly more reliable. We show that it is completely feasible to distill this high-quality label from the same EHR data, using a robust ML model.

The expertise required to implement an ML model might seem daunting in comparison to classical approaches such as chart review. The field of bioinformatics is rapidly expanding, and the ever-increasing offer of modeling techniques can come across as overly complicated. However, in previously published work, we introduce and validate a user-friendly pipeline for the construction of an ML model tailored to individual healthcare centers, requiring very limited knowledge of python [4]. There are no EHR or language-specific requirements to apply the pipeline. The relevant text should be extracted from the EHR and fed into the pipeline together with an annotated subset. The development of a standalone application is a planned future step. By relieving the need for ML-specific knowledge, we enable everyone with access to an annotated set of cases to create classifiers on par with the SVM model presented here [3].

### Limitations

In our study, we compare the ML extracted rheumatologist’s diagnosis with the criteria-based RA identification in one center. It remains unknown whether rheumatologists at other centers would have selected a similar RA population. For future international multi-center research, we would need to compare the differences in the patient characteristics of different centers and investigate the effect of EHR language on the model performance. The pipeline employed here for the construction of the ML model is language-independent and has been previously shown to perform well in both Dutch and German through the use of external lemmatization packages from NLTK (Natural Language Toolkit) [16] to increase the quality of the preprocessing procedure. To further aid the generalizability, our pipeline compares different ML techniques to deduce the best fit on the local data. Further studies into the consistency of our findings go beyond the scope of the current study.

Our study is not the first to apply machine learning and natural language processing to extract information from EHR free-text narratives. Recent years have seen many ML systems for various clinical applications. Specific tools have been developed to extract the diagnosis, symptoms, or to predict the prognosis and/or treatment response [17, 18]. Lin et al. (2013) built a classifier to predict the disease activity of RA patients, but had to complement narrative data with the ESR and CRP [19]. It is important to note that most of these methods in the literature provided one fixed algorithm that only fit a certain language or EHR system. Therefore, we developed a generalizable pipeline by which one can build an EHR-specific algorithm, which is not restricted to one language or EHR software [4].

While we emphasize the very sensitive nature of our approach as a strength, therein lies a seeming limitation as well; case selections made using the initial SVM model (cutoff 0.83) will be diluted by non-cases to a larger extent than the stringent classification selections. However, as we have described in our methodology, we choose to optimize the trade-off between sensitivity and PPV, which results in a particular cutoff point for the binarization of the SVM probabilities. This threshold can be modified to fit one’s research purposes, for example, optimizing the specificity only or the balance between specificity and sensitivity (Figure S1 and Table S1 and S2). This flexibility opens this methodology up to a wide field of potential applications.

## Conclusion

Using ML methods to extract the physician opinion from free written text, recorded as part of standard clinical care, allows for a sensitive collection of cases with clinical manifestations similar to traditional, criteria-based, and selection of patients. This approach for high throughput identification of disease case selections will be invaluable in research into the larger entity of disease. Including the widest range of trustworthy cases is crucial when, for example, looking into novel patient subgroups within a disease or when identifying novel risk and susceptibility factors for complex illnesses.

## Supplementary Information

**Additional file 1.** Supplementary Table 1. Comparison of baseline characteristics between the stringent ML defined cohort (cutoff=0.99) and the two criteria based cohorts.

**Additional file 2.** Supplementary figure 1 Swarm plot SVM depicting the support vector machine–derived probability of being either non-rheumatoid arthritis (blue) or rheumatoid arthritis (green) in the model development set. The dotted lines display the optimal cutoffs: 0.99 (PPV>0.95), 0.83 (Sens>0.85; PPV>0.95) and 0.53 (Sens>0.95). Sens: sensitivity, Spec: specificity; PPV: positive predictive value; NPV: negative predictive value; Acc: accuracy; F1: F1 score. This figure is adapted from “Machine Learning Electronic Health Record Identification of Patients with Rheumatoid Arthritis: Algorithm Pipeline Development and Validation Study” by T.D. Maarseveen et al, 2020, JMIR.

**Additional file 3.** Supplementary figure 2 Upset plot visualizing the intersections of the ML defined cohort with the stringent cutoff (0.99) and the 2 criteria based gold standards, with a bar chart depicting the total cohort size in the bottom-left. Where C1987 = 1987 criteria based cases; ML= Machine learning based cases; C2010 = 2010 criteria based cases. N = 518 unique cases out of 1,127 records.

**Additional file 4.** Supplementary Table 2. Comparison of baseline characteristics between the lenient ML defined cohort (cutoff=0.99) and the two criteria based cohorts.

**Additional file 5.** Supplementary figure 3 Upset plot visualizing the intersections of the ML defined cohort with the lenient cutoff (0.53) and the 2 criteria based gold standards, with a bar chart depicting the total cohort size in the bottom-left. Where C1987 = 1987 criteria based cases; ML= Machine learning based cases; C2010 = 2010 criteria based cases. N = 565 unique cases out of 1,127 records.

## Data Availability

In accordance with the FAIR principles, we have made the machine learning scripts publicly available at GitHub [3]. The corresponding study data are available from the corresponding author upon reasonable request.

## Abbreviations

AUC-PRC: Area under the precision-recall curve
AUC-ROC: Area under the receiver operating characteristic curve
EHR: Electronic health record
ML: Machine learning
NPV: Negative predictive value
PPV: Positive predictive value
RA: Rheumatoid arthritis
SVM: Support vector machine
