# Supplementary material for: Handwork vs machine: a comparison of rheumatoid arthritis patient populations as identified from EHR free-text by diagnosis extraction through machine-learning or traditional criteria-based chart review
Source: Arthritis Res Ther. 2021 Jun 22;23:174. doi: 10.1186/s13075-021-02553-4 (PMC8218515; doi:10.1186/s13075-021-02553-4)
Supplement: Supplementary file 1 — Additional file 1. Supplementary Table 1. Comparison of baseline characteristics between the stringent ML defined cohort (cutoff=0.99) and the two criteria based cohorts. [file 13075_2021_2553_MOESM1_ESM.docx]

**Supplementary Table 1.** Comparison of baseline characteristics between the stringent ML defined cohort (cutoff=0.99) and the two criteria based cohorts.

|  | Predicted Case based on machine learning (cutoff=0.99) | 1987 Criteria Based Cases | 2010 Criteria Based Case |
| --- | --- | --- | --- |
| N☨ | 282 | 357 | 426 |
| Proportion Women | 0.66 | 0.63 | 0.66 |
| Proportion anti-CCP2 Positive | 0.58 | 0.49* | 0.48* |
| Proportion RF positive | 0.61 | 0.57 | 0.58 |
| Median DAS44 at Baseline | 2.8 | 2.9 | 2.9 |
| Median BMI | 26.0 | 25.5 | 25.5 |
| Median ESR | 28 | 29 | 27 |
| Median CRP | 9.0 | 10.2 | 9.0 |
| Median Age at Inclusion | 57.4 | 58.6 | 57.2 |
| Median Symptom Duration at Diagnosis (days) | 89.0 | 90.0 | 91.0 |
| Median Number of Swollen Joints | 5 | 6 | 6 |

*P-values were calculated with the Pearson Chi-Squared for proportions, Mann-Whitney U for medians: * p<0.05; ** p<0.01, *** p<0.001; ☨ Not statistically tested;*
