# Supplementary figures and images for: Handwork vs machine: a comparison of rheumatoid arthritis patient populations as identified from EHR free-text by diagnosis extraction through machine-learning or traditional criteria-based chart review
Source: Arthritis Res Ther. 2021 Jun 22;23:174. doi: 10.1186/s13075-021-02553-4 (PMC8218515; doi:10.1186/s13075-021-02553-4)

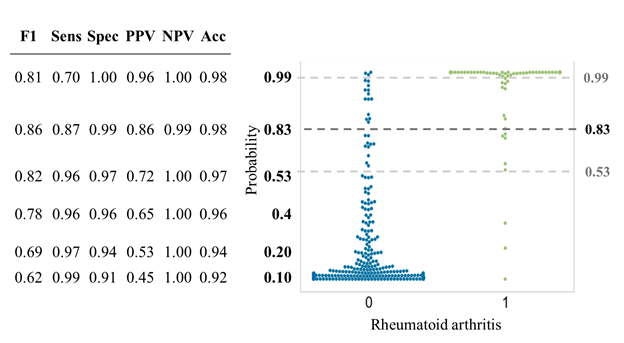

Supplement: Supplementary file 2 — Additional file 2. Supplementary figure 1 Swarm plot SVM depicting the support vector machine–derived probability of being either non-rheumatoid arthritis (blue) or rheumatoid arthritis (green) in the model development set. The dotted lines display the optimal cutoffs: 0.99 (PPV>0.95), 0.83 (Sens>0.85; PPV>0.95) and 0.53 (Sens>0.95). Sens: sensitivity, Spec: specificity; PPV: positive predictive value; NPV: negative predictive value; Acc: accuracy; F1: F1 score. This figure is adapted from “Machine Learning Electronic Health Record Identification of Patients with Rheumatoid Arthritis: Algorithm Pipeline Development and Validation Study” by T.D. Maarseveen et al, 2020, JMIR. [file 13075_2021_2553_MOESM2_ESM.png]

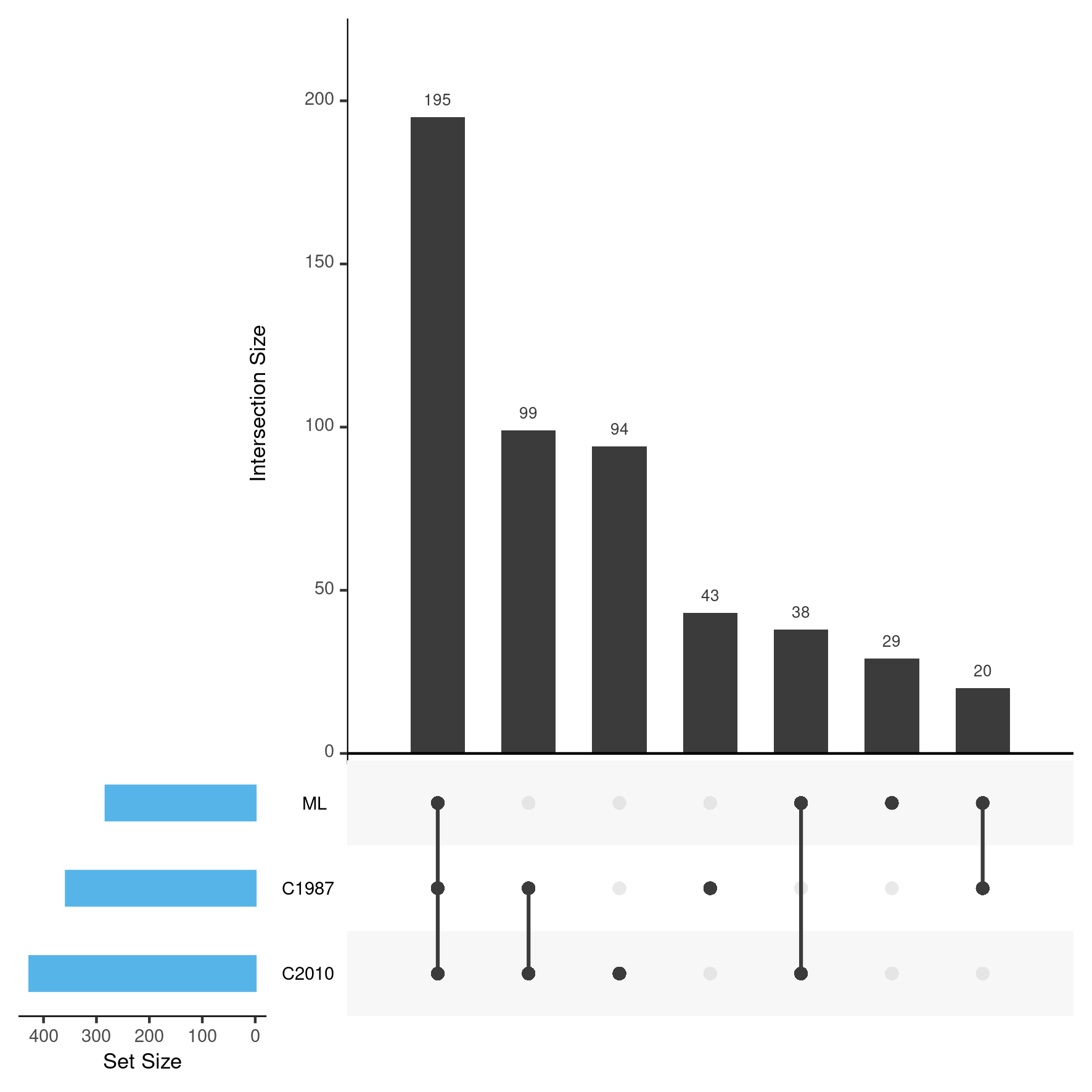

Supplement: Supplementary file 3 — Additional file 3. Supplementary figure 2 Upset plot visualizing the intersections of the ML defined cohort with the stringent cutoff (0.99) and the 2 criteria based gold standards, with a bar chart depicting the total cohort size in the bottom-left. Where C1987 = 1987 criteria based cases; ML= Machine learning based cases; C2010 = 2010 criteria based cases. N = 518 unique cases out of 1,127 records. [file 13075_2021_2553_MOESM3_ESM.png]

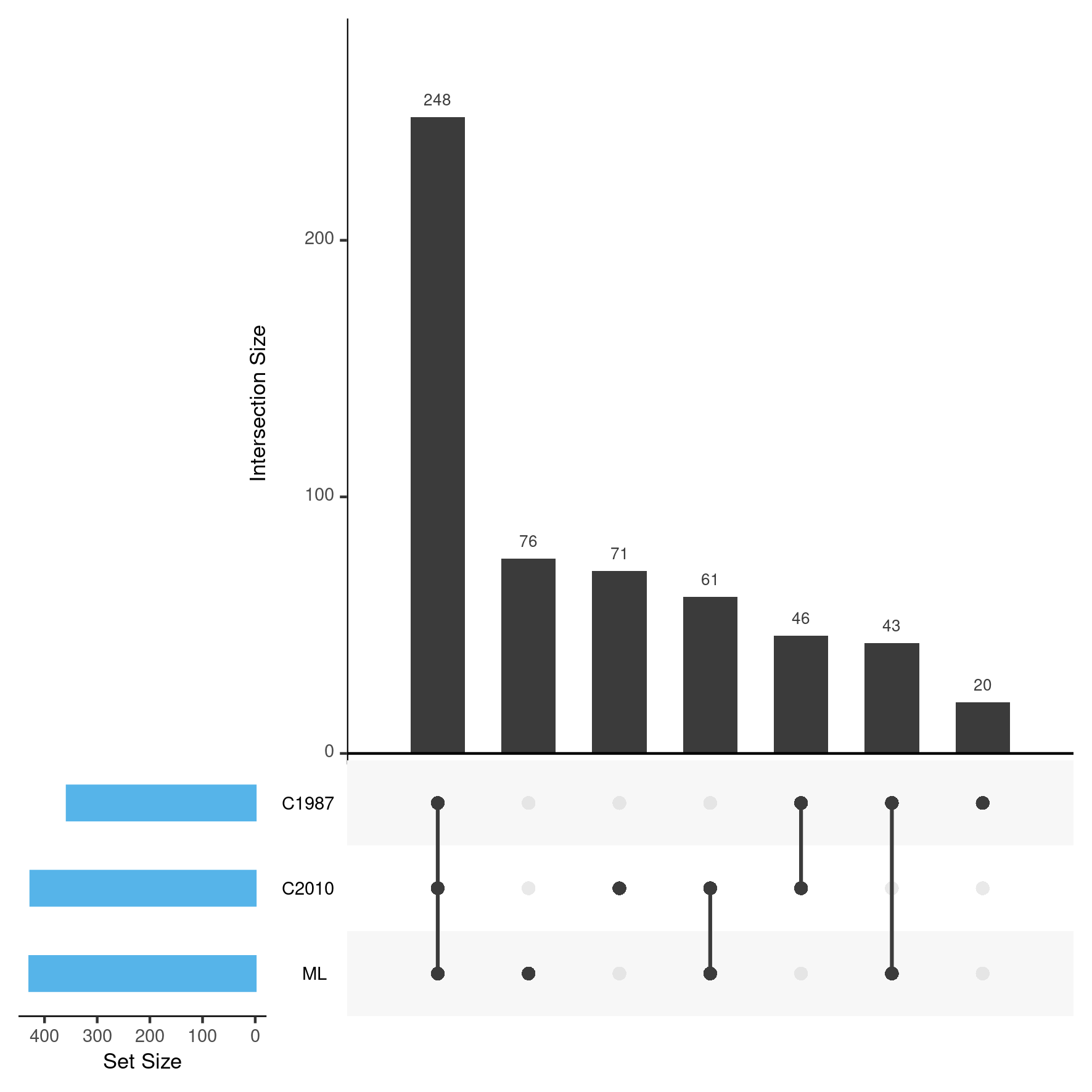

Supplement: Supplementary file 5 — Additional file 5. Supplementary figure 3 Upset plot visualizing the intersections of the ML defined cohort with the lenient cutoff (0.53) and the 2 criteria based gold standards, with a bar chart depicting the total cohort size in the bottom-left. Where C1987 = 1987 criteria based cases; ML= Machine learning based cases; C2010 = 2010 criteria based cases. N = 565 unique cases out of 1,127 records. [file 13075_2021_2553_MOESM5_ESM.png]
